# Effect of excess iodine intake on thyroid diseases in different populations: A systematic review and meta-analyses including observational studies

**DOI:** 10.1371/journal.pone.0173722

**Published:** 2017-03-10

**Authors:** Ryoko Katagiri, Xiaoyi Yuan, Satomi Kobayashi, Satoshi Sasaki

**Affiliations:** 1 Department of Social and Preventive Epidemiology, Graduate School of Medicine, the University of Tokyo, Bunkyo-ku, Tokyo, Japan; 2 Department of Social and Preventive Epidemiology, School of Public Health, the University of Tokyo, Bunkyo-ku, Tokyo, Japan; Institut de recherche pour le developpement, FRANCE

## Abstract

**Background:**

Although several reports concerning the association of iodine excess and thyroid disease have appeared, no systematic review of the association between iodine excess intake and thyroid diseases, especially hyperthyroidism and hypothyroidism, has yet been reported.

**Method:**

We conducted a systematic search of Ovid MEDLINE, PubMed, Cochrane Central Register of Controlled Trials databases, Ichushi-Web and CiNii database for intervention trials and observational studies. Search terms were constructed from related words for excess AND iodine intake or excretion AND thyroid hormones or diseases AND study designs. After considering the qualitative heterogeneity among studies, a meta-analysis was conducted and odds ratios and 95% confidence intervals (CI) were estimated in random-effects models. A protocol was registered with PROSPERO (No. CRD42015028081).

**Results:**

50 articles were included, including three intervention trials, six case-control studies, six follow-up studies and 35 cross-sectional studies. Three cross-sectional studies in adults included in meta-analysis. Odds ratio of overt and subclinical hypothyroidism between excess and adequate populations were 2.78 (CI:1.47 to 5.27) and 2.03 (CI:1.58 to 2.62) in adults, respectively. Source of excess iodine status was mainly iodized salt or water in included studies.

**Conclusion:**

Although universal salt iodization has improved goiter rates, chronic exposure to excess iodine from water or poorly monitored salt are risk factors for hypothyroidism in free-living populations. Monitoring of both iodine concentration in salt as well as the iodine concentration in local drinking water are essential to preventing thyroid diseases. Hypothyroidism should be also carefully monitored in areas with excess iodine. Because of the low quality and limited number of included studies, further evidence and review are required.

## Introduction

Iodine deficiency disorders are a major global public health problem. The World Health Organization (WHO) strongly recommends that “All food-grade salt, used in household and food processing, should be fortified with iodine as a safe and effective strategy for the prevention and control of iodine deficiency disorders in populations living in stable and emergency settings” [1]. Following the recommendation of the WHO and United Nations Children’s Fund (UNICEF) in 1993, universal salt iodization (USI) has been implemented in over 120 countries [2]. At the same time, monitoring iodine concentration in salt is recommended to prevent excess iodine intake [1]. Regarding iodine intake, the Tolerable Upper Intake Level (UL) for adults in the US is 1100 μg/day for adults [3]. However, instead of assessing iodine intake itself, measurement of urinary iodine concentration (UIC) or urinary iodine excretion per day (UIE) in a population is recommended as a reflection of recent iodine intake because “urinary iodine is well-accepted, cost-efficient and easily obtainable indicator for iodine status” [1]. Since it is easier to assess UIC than UIE, the WHO’s epidemiologic criteria define a median UIC ≥300 μg/L as “Excessive” in a population of school-age children (6 years or older). This cut-off value can be applied to adults, with the exception of pregnant or lactating women [4].

Excess iodine intake is considered to be associated with hyperthyroidism or hypothyroidism in some vulnerable individuals [5, 6]. Iodine-induced hyperthyroidism (IIH) has been reported as a side effect of iodine supplementation. This is also called as "Jod-Basedow phenomenon". IIH is likely to occur in individuals with thyroid nodular changes or in populations in whom iodine intake follows recent iodine fortification [7, 8]. Delange *et al*. described IIH in two African countries which had previously experienced severe iodine deficiency and had recently introduced iodized salt but with poor monitoring (median urinary iodine of 300–450 μg/L) [7–9]. In contrast, only a few papers have described IIH in iodine-sufficient countries around the world [10]. Among iodine-sufficient areas, IIH was initially reported in individuals living in Boston after administration of a high dose of iodine (180 mg per day) [11]. In Japan, a country with high iodine intake, two cases of IIH in women who consumed 28 mg–140 mg of iodine per day as soup stock from kelp was reported [12].

The other side effect of iodine excess is hypothyroidism. A reduction in thyroid hormones under a high iodide concentration is called the “Wolff-Chaikoff effect” [13]. Normally, thyroid hormone levels return to normal after a few days of this effect, termed the so-called “escape” or “adaptation” phenomenon [14]. Although the mechanism of iodine-induced hypothyroidism remains unclear, failure of this adaptation is considered to play a role. As is the case of IIH, individuals with predisposing thyroid damage such as autoimmune thyroiditis or thyroidectomy are susceptible to iodine-induced hypothyroidism [6].

Although several non-systematic reviews have examined the association between iodine excess and thyroid diseases [5, 6, 15], no systematic review has appeared to date. Here, therefore, we conducted a systematic review to summarize previous studies, mainly observational studies, because a randomized trial to examine the association between excess iodine and occurrence of thyroid diseases is considered to be difficult. Moreover, effect of chronic excess iodine exposure could be observed mainly in observational studies. On the contrary, acute excess or toxic amount of intake were not included in this review because the focus of this study was to identify the effect on thyroid under chronic iodine excess. The specific aims of the paper was (1) to confirm whether excess iodine is associated with thyroid diseases such as hyper- and hypo-thyroidism focusing on free-living populations; (2) to identify what kind of thyroid disease is likely to occur under chronic excess iodine status and (3) to find common features in thyroid hormone status in people with excess iodine intake.

## Methods

### Search strategy

Ovid MEDLINE, PubMed, Cochrane Central Register of Controlled Trials databases were searched for intervention trials and observational studies on the effect of excess iodine intake on thyroid diseases. Additionally, Ichushi-Web and CiNii database were used to search for relevant Japanese papers. *Ichushi*-Web and CiNii database were used for Japanese papers. The first search was conducted on 24 Nov 2015 and the latest search on 3 June 2016. A protocol of this review was registered with PROSPERO (No. CRD42015028081). Search terms were formulated as follows: related words for excess AND iodine intake or excretion AND thyroid hormones or diseases AND study designs (S1 Table). Although long term exposure of excess iodine were focused on in this review, search words were not limited because there was a possibility that words for exposure term did not appear in titles or abstracts. Language of publications was limited to English or Japanese. Only human studies were included, and year of publication was not limited. Two authors (RK and XY) independently screened the title and abstract for eligibility and then assessed the full text as below. Disagreements were resolved by discussion to reach consensus. This review was conducted according to Preferred Reporting Items for Systematic Reviews and Meta-Analyses (PRISMA) checklist. (S2 Table)

### Study selection

Eligibility criteria were original articles which examined the relationship between iodine exposure in excess and the incidence or prevalence of thyroid diseases, or between iodine excess and changes in thyroid hormones in free-living populations. Eligible populations were free-living adults (including apparently healthy elderly in nursing homes), adolescents, children and infants. A paper concerning newborns was excluded because the iodine status of newborns was considered to be influenced by their mother’s situation, and not reflective of the iodine intake of the newborns themselves. Eligible iodine exposure was excess urinary iodine excretion or excess iodine intake. Medications, radiation or other unnatural sources of iodine except oral supplementation were excluded in this systematic review.

Specific cut-off values for excess urinary iodine excretion were 300 μg/L in urinary iodine concentration in populations of school-age children and adults and 500 μg/L for pregnant women, in accordance with the WHO epidemiologic criteria for assessing iodine nutrition [4]. Since the WHO criteria do not define a cut-off value as “excess” for lactating women, the value of 500 μg/L was also used for lactating women. Similarly, 300 μg/L was used for children aged 2 to 5 years. If urinary iodine concentration was described in micrograms per gram creatinine (μg/g·Cre), based on urine and creatinine excretion of 1.5 L and 1 g per day, respectively, the cut-off for adults was defined as 450 μg/g·Cre (300 μg/L*1.5L). For children, based on urine and creatinine excretion of 500–1500 ml and around 20 mg/kg per day [16], and assuming that the value in μg/g·Cre is close to that in μg/L, the cut-off value for school-age children was defined as 300 μg/g·Cre. For pregnant women, lactating women and children under 6 years old, we decided to discuss inclusion criteria when any articles found during the search which used μg/g·Cre. Accordingly, eligibility criteria for excess iodine excretion were as follows: 1) papers which included populations with a median or mean urinary iodine concentration above the cut-off values; and 2) papers which included one population group (category) with a urinary iodine concentration above the cut-off values.

Intake in studies using dietary records, 24-hour recall or food frequency questionnaires were recognized as excess when the amount was over 1100 μg/d for adults, using the UL value in the US dietary reference intake [3]. Iodine supplementation was judged using the same value. Although the Institute of Medicine has set ULs of 300 μg/d for 4-8-year-olds, 600 μg/d for 9-13-year-olds and 900 μg/d for 14-18-year-olds, we decided to use 600 μg/d for populations under 18 years because papers sometimes included populations across several age ranges. For intervention trials, oral iodine tablet supplementation was included, while iodized salt or iodine-containing oil (eg. Lipiodol) were excluded. This is because total iodized salt intake was difficult to measure accurately by household survey, and oil was not typically used for continuous intake. Eligible outcomes were thyroid diseases (hyper- and hypo-thyroidism, goiter and nodule), thyroid volume and thyroid hormones (thyroid-stimulating hormone (TSH), triiodothyronine (T3), thyroxine (T4) and thyroglobulin (Tg)). Because we decided not to assess the association between antibody or autoimmune thyroiditis and excess iodine intake in this review, anti-thyroid antibody was included in search terms only, and was not included in eligible studies. We included studies with intervention (randomized and non-randomized trials), cohort, case-control, and cross-sectional designs.

### Quality assessment and data extraction

Data extraction and quality assessment of selected papers were conducted by one author (RK). The following information was extracted and tabulated: basic information, study design, setting, exclusion criteria, sample size, characteristics of participants, study period, assessment method of exposure and outcome, adjusted confounders and main findings, such as crude values, percentages and measures of association. If several adjusted models were described, measures of association were extracted from the fully adjusted model. Although diagnostic criteria of thyroid diseases differed slightly among studies, especially the cut-off value of thyroid hormones, data were obtained in accordance with the paper described. Contact with authors was not undertaken.

Regarding the study quality of randomized trials, risk of bias was assessed according to the domains in the Cochrane Handbook for Systematic Reviews of Interventions as follows: 1) random sequence generation, 2) allocation concealment, 3) blinding of participants and personnel, 4) blinding of outcome assessment, 5) incomplete outcome data, and 6) selective reporting [17]. Observational studies were assessed with the Risk of Bias Assessment tool for Non-randomized Studies (RoBANS), which includes the following six domains: 1) selection of participants, 2) confounding variables, 3) measurement of exposure, 4) blinding of outcome assessments, 5) incomplete outcome data, and 6) selective outcome domains [18] (S3 Table). The results from these assessments were used qualitatively.

### Data analyses

Meta-analysis was conducted after consideration of the number of included comparison studies and qualitative heterogeneity among studies for each outcome. Odds ratios and 95% confidence intervals (CIs) were calculated and meta-analysis was carried out using a random-effects model in Mantel-Haenszel analysis. The heterogeneity of studies was assessed qualitatively and quantitatively. Qualitative heterogeneity was considered in terms of population characteristics, year, country, setting, assessment method, implication and coverage of USI, and analysis method of outcome. Statistical heterogeneity was assessed with I^2^ and p-value in the chi-square test. An I^2^ more than 50% indicted the presence of heterogeneity [19].

## Results

**Fig 1:**
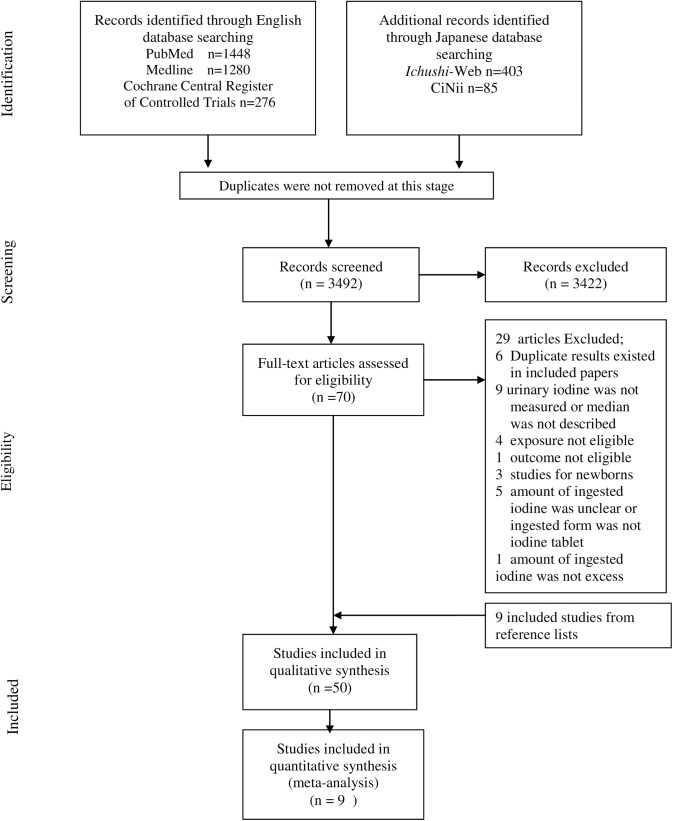
Flow chart of the selection process (searched on 3 June 2016).

After the screening of titles and abstracts, we identified 70 relevant articles (Fig 1). The full text of these articles was assessed and 41 were identified for inclusion. We hand-searched the titles of the references in these 41 included articles and examined the abstracts of potential papers. This step revealed a further nine eligible articles, giving a total of 50 papers for inclusion. Only one of these was identified from the Japanese databases. These 50 articles included three intervention trials (two randomized controlled trials and one non-randomized controlled trial), six case-control studies, and six follow-up (cohort) studies, including four studies from one survey. The remaining 35 papers were cross-sectional studies. Eight papers were conducted in adults, excluding pregnant women, 23 in children aged 6 months to 19 years (including one study which examined both adults and children), and five in pregnant women. Meta-analysis could not be carried out on the intervention, case-control or follow-up studies because the number of studies for each outcome was considered too small to integrate in meta-analysis. For the cross-sectional studies, meta-analysis was conducted only for overt hypothyroidism (OH) in adults and subclinical hypothyroidism (SCH) in adults, children and pregnant women. For hyperthyroidism and OH in children and pregnant women, the number of cases in each study was small, with most fewer than ten cases. We also excluded goiter and other outcomes from meta-analysis because we judged that these studies had substantial qualitative heterogeneity with regard to iodine source, coverage of USI and outcome assessment method. Sensitivity analysis was not conducted because of the small number of included papers.

### Results from intervention trials

The three selected intervention trials and their results are shown in Table 1 [20–22]. All three were in adults and intervention period was more than 4 weeks in all papers. Sang *et al*. conducted a double-blinded randomized controlled trial to explore the safe upper level of iodine intake for Chinese [20]. The quality of this study was judged to be medium. However, the other two papers [21,22] had problems in outcome reporting or study design. Although they differed with regard to baseline UIC, participant age and amount of administration, median or mean UIC and thyroid stimulating hormone (TSH) rose steeply at the time of supplement termination. During the follow-up period (2–4 weeks after the termination of supplementation), UIC returned to almost baseline level but TSH remained high in some participants. In China, iodine intake over 800 μg/d caused prolonged SCH in populations with “above requirement” baseline iodine status [20]. Hyperthyroidism was also observed on administration of 50 mg iodine in an elderly population with mild iodine deficiency at baseline [21].

**Table 1:** Intervention studies, including randomized controlled trials, of the impact of an excess or safety upper limit of iodine supplementation in thyroid function.

Author, Year, Country,Age	Intervention	Baseline	Post-intervention				Follow-up				Clinical outcome
			MUIC	TSH	FT4	FT3	MUIC	TSH	FT4	FT3	
Sang, 2012, China, 19-25y [20]	0–2000 μg/d iodine for 4 wk	• MUIC:237–381 μg/L • TSH:2–2.7mIU/L • FT4:16.2–19.1 pmol/L • FT3:4.7–5.3pmol/L • Thyroid volume:11.0–14.8 ml	• 4w • 1.5-fold increase in 200 μg/d • 3-fold in 500 μg/d • 5-fold in 750–1000 μg/d • 6-7-fold in 1250–2000 μg/d	• Increased by 20–60% in 0–400 μg/d • 51–109% in 500–2000 μg/d • All p<0.05	• Increased by 1–5% in 0–400 μg/d • Decreased by 3–8% in 500–1250 μg/d • Decreased by 12–17% in 1500–2000 μg/d p<0.05	• Changed by 2–4% in 0–400 μg/d • Decreased by 6–12% in 500–2000 μg/d p<0.05;					• At 4wk, from 300 μg/d, subclinical hypothyroidism appeared (5%), 18–47% in 750–2000 μg/d • After 1–3 month, 5–12% in 400–2000 μg/d
Thomson, 2011, New Zealand, mean 73y [21]	• >50 mg iodine as iodate/day for 8 weeks (high I; n = 21) • 80 μg iodine as iodate/day (low I; n = 25); • placebo(n = 24)	• MUIC: 54.5μg/L • TSH:2.6mIU/L • FT4:14.1pmol/L • FT3:4.86pmol/L	• 8w • 6-9-fold increase in high I	• 1.6-10-fold increase (high I; n = 4) • Decreased to 1/38-1/7 (high I;n = 3)	• Decreased by 11–50% (high I;n = 4) • Increasing by 71% (high I;n = 1)	• Increased by 52–54% (high I;n-2)	• Stop after 4w • 0.7–1.1-fold change in high I • 1.1–1.5-fold in low I	• Increased by 27–92% (high I;n = 2)Decreased to 1/7-1/114 (high I;n = 2)	• Increased by 60–91% (high I:n = 2)	• Increased by 28–65% (high I;n = 2)	• Transient subclinical hypothyroidism (n = 2) Transient clinical hypothyroidism (n = 2) • Hyperthyroidism (n = 2)
Namba, 1992, Japan, 25-39y [22]	After 1 wk restriction of iodine intake, 27 mg iodine/day for 4 wk	• Mean UIC: 43 nmol/μmol creatinine • TSH: 0.95 mU/L • FT4: 19.4 pmol/L • FT3: 1.84 nmol/L	• 4w • 30-fold increase	2.5-fold increase	Decreased by 15%	Not assessed	• Stop after 4 w • Returned to baseline level	Increased by 42%	Decreased by 5%		

Abbreviations: MUIC, median (or mean) urinary iodine concentration; TSH, thyroid-stimulating hormone; FT3, free triiodothyronine; FT4, free thyroxine

### Results from case-control studies

The six case-control studies are shown in Table 2 [23–28]. Cases were hypothyroidism in three [23, 26, 28], nodule in one [24], pregnancy in one [25] and several thyroid diseases including hyperthyroidism, hypothyroidism, thyroiditis and nodule in one [27]. All papers were in adults. Regarding subclinical and overt hypothyroidism, median or mean UIC in the case group was 300 μg/L and higher than that in the control group in three of the four studies. Only one study, from Japan, reported lower urinary iodine excretion in the hypothyroidism group [28]. With regard to quality assessment, although five of the six studies matched for mean age range (except Wang *et al*. [24]), none of the six adjusted their analysis for targeted diseases.

**Table 2:** Case-control studies, including populations with excess mean or median urinary iodine concentration (adults).

Author, Year, Country, Age	Case	Median or mean UIC (μg/l) Case, Control	TSH	FT4 (pmol/L)	FT3 (pmol/L)	Hyperthyroidism	Subclinical hyperthyroidism	Hypothyroidism	Subclinical hypothyroidism
Kotwal, 2015, India, mean 32y [23]	overt hypothyroidism (n = 150), hospital (n = 154) and community (n = 488) controls	Lower in control: 310 vs 301 (p = 0.02) vs 215 (p = 0.001)	13.2 vs 2.3 vs 2.3 mU/L p<0.05						
Wang, 2014, China, mean 49y [24]	benign thyroid nodule (n = 51); control (n = 306)	Higher in case: 331.33 vs 174.3 P<0.001	2.32 vs 2.28 μU/ml	17.4 vs 17.1	4.19 vs 4.2				
Du, 2013, China, mean 27y, 29y [25]	27wk after pregnancy (n = 300); control (n = 300)	1227.9 vs 951.2	2.9 vs 3.1 mU/l	13.5 vs 14.3 p< 0.01	4.0 vs 4.7 p< 0.01	0.3% vs 3% p<0.05	19.7% vs 27.3% p = 0.027	0.7% vs 0.3% p<0.05	2% vs 0.3% p<0.05
Alsayed, 2008, Egypt, mean 29y [26]	autoimmune subclinical hypothyroidism (n = 73); control (n = 60)	Higher in case: 326.97, 274.45 P<0.01	Higher in case: 8.29 vs 2.07 μU/ml p<0.001	9.29 vs 2.07 pmol/L					
Kim, 2000, Korea, mean 42y [27]	thyroid disease (n = 184); control (n = 207)	• single goiter (n = 17) mean 2880 NS • hyperthyroidism (n = 42) 4900 p<0.05 • hypothyroidism (n = 15) 4570 p<0.05 • subacute thyroiditis (n = 15) 4690 p<0.05 • painless thyroiditis (n = 12) 3460 NS • Hashimoto's thyroiditis (n = 36) 4140 p<0.05 • benign thyroid nodule (n = 36) 2950 NS • control (n = 207) 2110μg/day							
Ishizuki, 1992, Japan, 32-74y [28]	hypothyroidism (n = 8), chronic thyroiditis (n = 32), control (n = 32)	Lower in hypothyroidism group: 268.3 vs 471.8 vs 465.6 μg/day	75.3 vs 0.8 vs 1.2 μU/ml	T4 (μg/dl) 4.1 vs 9.1 vs 8.3	T3 (ng/dl) 112.8 vs 135 vs 132.4				

Abbreviations: MUIC, median or mean urinary iodine concentration; TSH, thyroid-stimulating hormone; FT4, free thyroxine; FT3, free triiodothyronine; NS, not significant

### Results from follow-up studies

Six papers from three surveys were included, including two from China and one from a refugee camp in Algeria (Table 3) [29–34]. Teng *et al*. [29] and three other papers [30–32] reported from three areas in China as follows: inhabitants consumed locally produced salt with low levels of iodine, even after salt iodization was begun in 1996 in the first area; iodized salt was used and iodine status of the population was improved from mildly deficient in the second area; and an excess iodine level owing to drinking water was reported in the third area. Wang *et al*. also reported the time course of changes in UIC and goiter rate before and after the implementation of universal salt iodization in China [33]. Teng *et al*. reported that the prevalence of OH and SCH was the highest in the excess area, and that excessive intake was a risk factor for SCH at follow-up among subjects who were normal at baseline, while a shift in iodine intake from mildly deficient to more than adequate was a risk factor for continued SCH [29]. Aakre *et al*. also reported changes in thyroid dysfunction in lactating women [34]. Although lactating status at baseline in this population might have changed by the time of follow-up, three-quarters of subjects with hypothyroidism retained their hypothyroidism at three-year follow-up while nearly 8% of subjects developed new subclinical hypothyroidism. In their logistic regression model, Teng *et al* found that both excess iodine intake and mildly deficient iodine intake were risk factors for goiter in normal subjects. [29]. Wang *et al*. reported that total goiter rate was correlated with average thyroid volume after the elimination of iodine deficiency diseases, and that an an increase in UIC from less than 300 μg/L to over 300 μg/L decreased the average thyroid volume whereas a steady state of over 300 μg/L was associated with a slight increase in the average thyroid volume [33].

**Table 3:** Follow-up studies including populations with excess mean or median urinary iodine concentration.

Author Year, Country, Age	Baseline							Follow-up						
	MUIC	Overt Hyperthyroidism	Subclinical hyperthyroidism	Overt hypothyroidism	Subclinical hypothyroidism	Goiter	Nodule	MUIC	Overt Hyperthyroidism	Subclinical hyperthyroidism	Overt Hypothyroidism	Subclinical hypothyroidism	Goiter	Nodule
Teng, 2006, China, >13y [29] ^a^	615 (A) vs 375 (B) vs 103 (C)	1.2% vs 2.0% vs 1.6%	• 1.1% vs 3.9% vs 3.7% • Adjusted OR 0.22 (A vs C (ref)) [31]	2% vs 0.9% vs 0.3%	• 6.1% vs 2.9% vs 0.9% • Adjusted OR 6.39 (A vs C (ref)) [31]	7.6% vs 16.9% vs 23.2%	10.8% vs 10.2% vs 12.6%	• 5 year • 635 vs 350 vs 97	• 5y incidence • 0.8% vs 0.9% vs 1.4%	1% vs 2% vs 1.4%	0.3% vs 0.5% vs 0.2%	• 2.9% vs 2.6% vs 0.2% • OR 9.1 (A vs B,C normal to SCH)	• 7.7% vs 6.8% vs 12.1% • OR 1.46 (A vs B (ref)) [30]	6.6% vs 6.9% vs 4.4%
Wang, 2015, China, 8-10y [33]	83 μg/L in 1995, 377 μg/L in 1997 (n = 4767)					55.2% in 1997		407 μg/L in 1999, 334 μg/L in 2001; 270 μg/L in 2002, 243 μg/L in 2005; 325 μg/L in 2009, 345 μg/L in 2011					23.3% in 1999; 2.9% in 2001; 3.9% in 2002; 3.8% in 2005; 1.0% in 2009; 1.7% in 2011	
Aakre, 2015, refugee camps [34] Baseline lactating women	350 μg/L (n = 111)	5.4%	2.7%	4.5%	14.4%			• 3 year • 617 μg/L (n = 78)	3.0%	4.5%	1.5%	22.4%		

^a^ Papers in reference nos. [30] [31] and [32] were from the same reference dataset [29]. In the article [32], thyroglobulin (Tg) was compared and shown to significantly differ among three areas. At baseline, median serum Tg (ng/mL) was 6.6 vs 5.6 vs 7.8 in area (A) vs (B) vs (C) in the table and 9.7 vs 8.6 vs 11.2 at follow-up.

Abbreviations: MUIC, median or mean urinary iodine concentration; OR, odds ratio; ref, reference

### Results from cross-sectional studies

Eight studies in adults were included [35–42]. Since only one of these reported biomarker values [36], Table 4 tabulates results for thyroid diseases only. The paper by Szabolcs *et al*. was in elderly subjects living in a nursing home [37]. Excess iodine intake was caused by high iodine content in drinking water in five papers [35–37, 41, 42], USI in one paper [37] and seaweed consumption in one paper [38]. The cause was not assessed in one paper because of an adequate median UIC [40]. Four studies compared high and low iodine status populations [35, 36, 37, 41], three papers described one population with a median or mean UIC of the total population of over 300 μg/L [38, 39, 42], and one paper reported that median UIC was adequate but assessed categories of UIC over 300 μg/L [41]. Regarding study quality, three papers used a regression model [35, 40, 42], of which two used models for some of the outcomes [35, 42].

**Table 4:** Cross-sectional studies including excess median urinary iodine in adults.

Author, Year, Country, Age	MUIC	Overt Hypothyroidism	Subclinical hypothyroidism	Hyperthyroidism (overt+subclinical)	Subclinical hyperthyroidism	Goiter	Nodule	Thyroid volume (ml)	Others
Du, 2014, China, >18y [35]	750.18 μg/L (excess group n = 930); 228.70 μg/L (sufficient group n = 550)	2.6% vs 1.2%	• Higher in excess: OR 11.7 (excess) vs 5.79 (sufficient) (vs ref p<0.01) • 20.1% vs 10.4%	• OR 0.404 (excess) vs 0.401 (sufficient) (excess p<0.005) • 1.2% vs 1.0%		• OR 0.981 (excess) vs 0.979 (sufficient) (vs deficient NS) • 2.5% vs 2.3%	• Higher in excess: OR 1.865 (excess) vs 1 (sufficient ref) p<0.01 • 15.5% vs 8.7%		
Tan, 2014, China, 20-50y [36]^a^	1152 μg/L (excess area n = 506); 185 μg/L (adequate area n = 348)	3.6% vs 1.3% NS	Higher in excess: 13.6% vs 9.0% p = 0.046	3.4% vs 1.3% NS	2.2% vs 0% NS				
Szabolcs, 1997, Hungary, 61-97y [37]	513 μg/g cre (n = 92 abundant); 100 μg/g cre (n = 135 prophylaxis)	7.6% vs 1.5% (vs deficient p = 0.006)	23.9% vs 10.4% (vs deficient p<0.001)	0% vs 3% NS	0% vs 1.5% NS	12.2% vs 16.4% (vs deficient 39.4% p<0.001)	3.3% vs 16.2% (vs deficient 20.2% p = 0.001)	15.1 vs 13.6 (vs deficient 21.9 p = 0.003)	
Konno, 1993, Japan, mean 45y [38]	27.1 μmol/L (3441μg/L n = 4110)	0.39%	1%	0.61%	0.27%				Graves’ disease 0.39% Hashimoto’s thyroiditis 8.1%
Gomo, 1999, Zimbabwe, >35y [39]	560 μg/L (n = 516)			3%					
Chen, 2013, China >18y [40]	All 172 μg/L (n = 9412); 100-<200 μg/L n = 3428; 200-<300 μg/L n = 2061 (high); ≥300 mg/L n = 1694 (excess)						Adjusted OR:1.01 (high) vs 0.97 (excess) (vs ref normal NS)		
Kassim, 2013, Somalia 15-49y women [41]	316 μg/L (n = 219 Zone A); 228 μg/L (n = 196 Zone B)					1.4% vs 3.3% (visible A vs B)			
Henjum 2011, refugee camp, 15-45y women [42]	466 μg/L (n = 388)					22%	16% (<1cm) 5% (>1cm)	Median 9.4	

^a^ In the paper by Tan *et al*. [36], serum FT4, FT3 and TSH values were compared between two areas. No significant differences were found in FT4 and FT3, while two values of TSH were significantly different. (Excess area vs adequate area, FT4 (pmol/l):14.7 vs 14.8, FT3 (pmol/l):4.8 vs 4.9, TSH (mIU/l) 2.7 vs 2.4)

Abbreviations: MUIC, median or mean urinary iodine concentration; OR, odds ratio ref; reference NS, not significant

Twenty-three papers reported cross-sectional studies in children [41, 43–64], of which 14 papers mainly reported thyroid diseases [41, 46, 47–49, 51–55, 56–58, 60], four reported biomarkers [61–64] and five described both [43–45, 50, 59] (Tables 5 and 6). Except for Nepal *et al*. in infants [43], most studies were in school-age children. Water was a cause of excess iodine status in ten papers, from China [44–48, 52, 58, 59], refugee camps in Africa [53] and Somalia [41]. USI caused excess intake in five papers, from Sudan [51], Saudi Arabia [49], Uganda [56], refugee camps in Africa [55] and Brazil [54]. Fortified food might have been a cause in two papers, from Sudan [51] and the USA [60], while seafood consumption might have been the cause in one paper from Japan [57]. The cause was not clearly described in others [43, 50] or was not assessed because median UIC was not excessive [61–64].

**Table 5:** Cross-sectional studies including excess median urinary iodine in children (outcome: thyroid diseases).

Author, Year, Country, age	MUIC	Overt Hypothyroidism	Subclinical hypothyroidism	Hyperthyroidism (overt+subclinical)	Goiter	Nodule	Thyroid volume (ml)
Nepal, 2015, Nepal, 0.5-2y [43]	407 μg/L (n = 630) 100–299 μg/L n = 91 (sufficient); ≥300 μg/L n = 375 (excess)	1% (sufficient) vs 0.8% (excess) NS	7.6% (sufficient) vs 7.4% (excess) NS	0% (sufficient) vs 1.5% (excess) NS			
Sang, 2013, China, 7-13y [44]	1030 μg/L (n = 371 high iodine area HI); 123 μg/L (n = 150 adequate iodine area AI)	1.1% (HI) vs 0% (AI)	Higher in HI: 6.7% (HI) vs 0.7% (AI) p = 0.004; Adjusted OR 3.62 (UIC≥600), 5.35 (UIC≥800)	2.7% (HI) vs 0.7% (AI) NS			
Gao, 2004, China, 6-11y [45]	Mean 631μg/L (n = 112) vs 338 μg/L (n = 110) vs 99 μg/L (n = 116)	0.9% vs 0% vs 0% NS	Higher in excess: 20.5% vs 14.6% vs 4.3% p = 0.001	0.9% vs 0.9% vs 1.8% overt NS subclinical NS			
Lv, 2014, China, 8-10y [46]	511 μg/L (n = 326 high iodine towns); 401 μg/L (n = 60 control town);				24.6% vs 14.0% (age-specific p = 0.015); 33.0% vs 17.5% (body surface area-adjusted p = 0.001)		
Kassim, 2013, Somalia, 6-11y [41]	398 μg/L (n = 268 Zone A); 288 μg/L (n = 239 Zone B)				0.3% vs 1.3% (visible A vs B)		
Lv, 2012, China, 8-10y [47]	418.8 μg/L (n = 363 one province)				11.0% (n = 1259)		
Li, 2012, China, 8-10y [48]	336.3 μg/L (n = 379 iodine in water 150–300μg/L); 494.8 μg/L (n = 173 iodine in water >300μg/L);				8.0% (iodine in water >150μg/L n = 550)		
Alsanosy, 2012, Saudi Arabia, 6-13y [49]	Median 421 μg/L (n = 311)				11% (palpation)		
Medani, 2012, Sudan, 6-12y [50]	464 μg/L (n = 654)				34.9% (palpation)		
Hussein, 2012, Sudan, 6-12y [51]	Median 553 μg/L (n = 140 high iodine city); 160 μg/L (n = 140 control city)				17.1% vs 1.4% NA (palpation)		
Shen, 2011, China, 8-10y [52]	460.9 μg/L (n = 24407 iodine in water 150–199 μg/L); 479.5 μg/L (n = 16940 iodine in water 200–249 μg/L); 644.4 μg/L (n = 11486 iodine in water 250–299 μg/L); 765.0 μg/L (n = 1882 iodine in water 300–349 μg/L); 919.4 μg/L (n = 515 iodine in water 350–399 μg/L); 791.3 μg/L (n = 547 iodine in water 400–499 μg/L); 969.8 μg/L (n = 974 iodine in water >500 μg/L)				• 6.2% (water 150–199 μg/L); 5.6% (water 200–249 μg/L); 7.6% (water 250–299 μg/L); 8.8% (water 300–349 μg/L); 11.1% (water 350–399 μg/L); 10.1% (water 400–499 μg/L); 15.8% (water >500 μg/L) • RR:1.56 (300–399 μg/L), 2.27 (600–699 μg/L), 3.46 (900–999 μg/L), 3.69 (>1500 μg/L) (ref 100–200) NA		
Henjum, 2010, refugee camp, 6-14y [53]	565 μg/L (n = 421)				56.2% (Tvol-for age>P97); 85.6 (Tvol-for-BSA >P97)		Median 5.0
Duarte, 2009, Brazil, 7-12y [54]	484.2 μg/L (boys n = 480); 435.3 μg/L (girls n = 484)				1.9% (1.6% boys, 2% girls >P97)	0.2% (0.4% boys, 0% girls)	5.43 (boys n = 480); 5.62 (girls n = 484)
Seal, 2005, 6 refugee camps in Africa, 10-19y [55]	Uganda 726μg/L Ethiopia 1074μg/L Algeria 1170μg/L Zambia 570μg/L				Uganda 0.4% Ethiopia 1.3% Algeria 7.1% Zambia 0% (visible)		
Bimenya, 2002, Uganda, 6-12y [56]	310 μg/L (n = 300)				60.2% ((palpation) down from 74.3 in 1991), visible goiter 30% (n = 2880)		
Ishigami, 2001, 2 countries, 7-17y [57]	Median 47.3 μg/L (n = 100 Belarus radio-contaminated area); 362.9 μg/L (n = 250 Japan)				13.6% (Belarus) vs 1.6% (Japan)	1.74% (Belarus) vs 0% (Japan)	
Zhao, 2000, China, 6-15y [58]	Median 520–1961 μg/L (12 townships, n = 607)				12–38% (palpation)		Abnormal 5–17%
Boyages, 1989, China 7-15y [59]	1236.5 μg/g cre (n = 29 excess area); 428.4 μg/g cre (n = 26 sufficient area)				65% (excess) vs 15% (sufficient) (palpation)		
Trowbridge, 1975, USA, 9-16y [60]	452 μg/g cre (n = 754)				6.8% (n = 7785) (palpation)		

Abbreviations: MUIC, median or mean urinary iodine concentration; OR, odds ratio; ref, reference; RR, relative risk; NA, not assessed in the study; NS, not significant

**Table 6:** Cross-sectional studies including excess median urinary iodine in children (outcome: biomarkers for thyroid hormone).

Author, Year, Country, Age	MUIC	TSH (mIU/L)	FT4 (pmol/L)	FT3 (pmol/L)	Tg (μg/L)	Thyroid volume (ml)
Shakya, 2015, Nepal, 6-11y [61]	All:292 μg/L (n = 640) 100–199 μg/L n = 126, 200–299 μg/L n = 148, >300μg/l n = 313	Median 3.5 (adequate) vs 3.7 (more than adequate) vs 3.2 (excess)	Median 16.6 vs 15.6 vs 15.9	Medianl 4.0 vs 3.6 vs 4.1	Median 13.7 vs 15.2 vs 10.9 (p = 0.016 vs 5 UIE categories) Mean 15.3 vs 18.6 vs 17.2	
Nepal, 2015, Nepal, 0.5-2y [43]	407 μg/L (n = 630) 100–299 μg/L n = 91 (sufficient), ≥300 mg/L n = 375 (excess)	Geometric mean: 2.9 (sufficient) vs 2.9 (excess)	Mean 16.8 (sufficient) vs 17.2 (excess) NS		Geometric mean:20.8 (sufficient) vs 21.9 (excess) NS	
Zou, 2014, China, 8-10y [62]	All: 173.3μg/L; 100–300 μg/L n = 56, >300 μg/l n = 38	Median 2.9 (excess) vs 2.8 (sufficient)	Median 18.7 (excess) vs 18.3 (sufficient)	Median 6.1 (excess) vs 6.0 (sufficient)		Median 3.13 (excess boy) vs 3.23 (sufficient boy); 3.85 (excess girl) vs 2.92 (sufficient girl)
Sang, 2013, China, 7-13y [44]	1030 μg/L (n = 371 high iodine area HI);123 μg/L (n = 150 adequate iodine area AI)	• Sensitive TSH • Higher in HI Median 4.01 vs 3.42 p = 0.001	Mean 16.4 (HI) vs 16.3 (AI)	Mean 6.28 (HI) vs 6.31 (AI)		Higher body surface area-adjusted Tvol in higher UIC beta = 0.22; P = 0.002
Zimmermann, 2013, 12 countries, 6-12y [63]	All: 151 μg/L (n = 2512 12 countries) 100–199.9 μg/l n = 609, 200–299.9 μg/l n = 468, >300 μg/l n = 477	Higher in more than adequate or excess; Mean 0.84 vs 0.87 vs 0.91 p<0.05			Highest in excess: Mean 9.4 vs 11.8 vs 17.4 p<0.05	
Medani, 2012, Sudan, 6-12y [50]	452.9 μg/L (n = 31 high iodine city), 51 μg/L (n = 329 other cities)	Higher in excess: Mean 3.71 vs 2.11 p = 0.008			Mean 46.0 ng/ml vs 37.2 ng/ml P = 0.052	
Zimmermann, 2005, 5 countries, 6-12y [64]	All 218 μg/L (n = 3319 7 areas) 728 μg/L (n = 280 highest iodine area)					All 2.54; 4.91 (highest area) age and body surface area adjusted; began to rise at UIC>500 μg/L
Gao, 2004, China, 6-11y [45]	Mean 631 μg/L (n = 112) vs 338 μg/L (n = 110) vs 99 μg/L (n = 116)	3.4 vs 3.3 vs 2.3 p = 0.02			13.7 vs 7.7 vs 3.2 p = 0.001	
Boyages, 1989, China 7-15y [59]	1236.5 μg/g cre (n = 29 excess area); 428.4 μg/g cre (n = 26 sufficient area)	Mean 5.2 (excess) vs 3.9 (sufficient) NS	Mean 19.1 (excess) vs 16.2 (sufficient) p<0.05			

Abbreviations: MUIC, median or mean urinary iodine concentration; TSH, thyroid-stimulating hormone; FT4, free thyroxine; FT3, free triiodothyronine NS, not significant

Five studies in pregnant women were identified (Table 7) [65–69]. Gestational age was first trimester in two studies [66, 69], third trimester in one [68] and all terms in two [65, 67]. Median UIC was less than 300 μg/L in two papers [66, 67], above 300 μg/L in two papers [65, 69], while excess and adequate iodine areas were compared in one paper [68]. Two papers included multivariate analysis [66, 67].

**Table 7:** Cross-sectional studies including excess median urinary iodine in pregnant women.

Author,Year, Country	Number of subjects, gestation	MUIC	Overt hypothyroidism	Subclinical hypothyroidism	Isolated hypothyroxinemia	Hyperthyroidism (overt and subclinical)	TSH mIU/L	FT4	FT3	Tg
Cho, 2015, Korea [65]	344, first-third trimester	Total: 427.3 μg/L 150–249 μg/L n = 47 (14%), 250–499 μg/L n = 74 (21%), ≥500 μg/L n = 150 (44%)					Median 1.44 (150–249 μg/L), 1.48 (250–499 μg/L), 1.56 (≥500 μg/L) NS			
Shi, 2015, China [66]	7190, 4-8w	Total: 152.6 μg/L; 150–249 μg/L n = 2459 (34.2%), 250–499 μg/L n = 1040 (14.5%), ≥500 μg/L n = 229 (3.2%)	No statistically significant differences were noted 0.7% (150–249 μg/L) 1.2% (250–499 μg/L) 0.9% (>500 μg/L)	Higher in excess: Adjusted OR 1 (150–249 μg/L ref), 1.72 (1.13–2.61) (250–499 μg/L), 2.17 (1.13–4.19) (≥500 μg/L) P<0.05; 2.4% 150–249 μg/L; 4.2% (250–499 μg/L); 5.7% (≥500 μg/L)	Higher in excess: OR 1 (150–249 μg/L ref), 1.05 (0.59–1.87) (250–499 μg/L), 2.85 (1.40–5.81) (≥500 μg/L)		Higher in excess: median 1.86 (150–249 μg/L ref), 2.07 (250–499 μg/L) P<0.001, 2.32 (≥500 μg/L) p<0.001	Lower in excess: median16.12 pmol/L (150–249 μg/L ref), 15.95 (250–499 μg/L) p = 0.06, 15.27 (≥500 μg/L) p<0.001		Higher in excess: median 10.18 μg/L (150–249 μg/L ref), 10.97 (250–499 μg/L) p = 0.001, 13.58 (≥500 μg/L) p<0.001
Habimana, 2014, Congo [67]	225, first-third trimester	Total: 138 μg/L; 150–249 μg/L n = 35 ≥250 μg/L n = 70	17% (150–249 μg/L) vs 1.4% (≥250 μg/L) P<0.01	9% (150–249 μg/L) vs 7% (≥250 μg/L)	Not statistically different		• 2.00mIU/L (150–249 μg/L) vs 1.42 (250 μg/L) • High TSH Adjusted OR 0.44 (250 μg/L, ref 150–249 μg/L)	Median 0.79 ng/dl (150–249 μg/L) vs 0.85 (250 μg/L)	Median 1.30pg/ml (150–249 μg/L) vs 1.38 (250 μg/L)	Median 13.0ng/ml (150–249 μg/L) vs 12.3 (250 μg/L)
Sang, 2012, China [68]	384, third trimester	1240.7 μg/L (n = 210, excess area), 217.06 (n = 174 adequate area)	0.5% vs 0%	20% vs 2.3% p<0.001 OR 6.2 (p = 0.04 UIC>250 ref UIC<250)		0.5% vs 0% (overt) 1.9% vs 0% (subclinical)	• Sensitive TSH • Higher in excess: mean 2.89 vs 2.19 p = 0.001	Lower in excess: mean 13.35 vs 13.77 p = 0.04	Higher in excess: mean 4.03 vs 3.78 p<0.001	
Orito, 2009, Japan [69]	514, 7-15w	328 μg/L					Higher in high urinary iodine r = 0.1326; p = 0.003	Lower in high urinary iodine r = -0.1801; p = 0.00004	Lower in high urinary iodine r = -0.1701; p = 0.00011	

Abbreviations: MUIC, median or mean urinary iodine concentration; TSH, thyroid-stimulating hormone; FT4, free thyroxine; FT3, free triiodothyronine; NS, not significant; r, correlation coefficient

### Hypothyroidism in cross-sectional studies in adults, children and pregnant women

Regarding OH and SCH, four studies in adults (Table 4) [35–38], three in children (Table 5) [43–45], and three in pregnant women (Table 7) [66–68] described the percentage or odds ratio of hypothyroidism.

**Fig 2:**
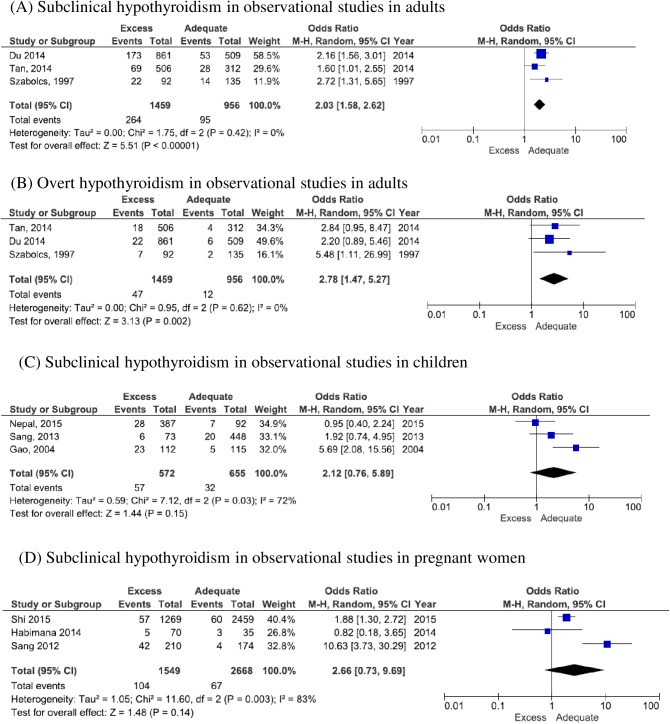
Forest plots of hypothyroidism in excess iodine status in observational studies. Crude number of cases and total was used to calculate odds ratio in each study. Adults [35–37], children [43–45], and pregnant women [66–68] were included in this analyses.

In the adult studies, the sex ratio of participants (male:female) was 1:2 to 1:3. The percentage or odds ratio of SCH was significantly higher in the excess group than in adequate group in most of these adult studies [35–37]. Forest plots for SCH and OH are shown in Fig 2A and 2B. Although one paper was for elderly participants [37], odds ratio in adults for OH and SCH were 2.78 (CI:1.47 to 5.27) and 2.03 (CI:1.58 to 2.62), respectively and when the study in the elderly was excluded, odds ratios were 2.44 (CI:1.21 to 4.91) and 1.95 (CI:1.47 to 2.58).

**Fig 3:**
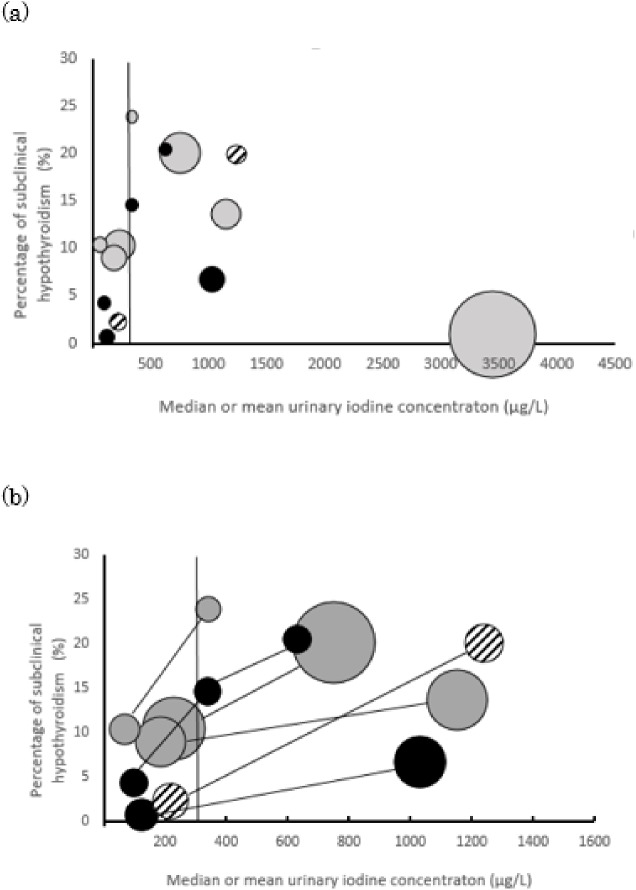
Plots regarding the prevalence of subclinical hypothyroidisim and urinary iodine concentration. **(a)** Size of a bubble shows the sample size of the study population. Gray bubbles are from studies in adults [35–38], black bubbles are for children [44, 45] and white with black line is for pregnant woman [68]. Papers which showed both the percentage of subclinical hypothyroidism and median or mean urinary iodine concentration were included. Papers which described UIC in categories and did not show the median or mean was excluded from this figure [43, 66, 67]. Vertical line of 300 μg/L is shown. **(b)** papers included in plot (a) and those compared in more than two areas in the study were plotted. Bubbles from the same paper were connected with lines. Colors of bubbles mean the same as (a).

In the child studies, the sex ratio of participants (boy:girl) was 1.2–1.5:1. The Forest plot for SCH in children is shown in Fig 2C, but heterogeneity was high. Heterogeneity was also high in pregnant women (Fig 2D). A bubble and spaghetti plot between urinary iodine concentration and the percentage of SCH in all age groups is shown in Fig 3. Papers which described UIC in categories and did not show the median or mean was excluded from this figure, because median or mean UIC could not be plotted [43, 66, 67]. Apart from one study from Japan [38], these studies show an increase in the percentage of SCH along with an increase in median UIC. Funnel plots of cross-sectional studies were not asymmetrical, albeit that the number of papers was limited (plots not shown).

### Hyperthyroidism in cross-sectional studies in adults, children and pregnant women

Hyperthyroidism was described in five studies in adults (Table 4) [35–39], three studies in children (Table 5) [43–45] and one study in pregnant women (Table 7) [68]. Iodine-induced hyperthyroidism is of concern where iodine supplementation has recently been introduced, but only one adult study, from Zimbabwe, met our inclusion criteria. The percentage of subjects with biochemical hyperthyroidism was 3% in this population [39]. Other studies compared more than two areas, one of which was an excess iodine area, but none of these studies showed a significant difference in adults, children and pregnant women studies.

### Goiter in cross-sectional studies in adults, children and pregnant women

The percentage of goiter was assessed in four adult studies (Table 4) [35, 37, 41, 42] and 16 studies in children (Table 5) [41, 46, 47–55, 56–60]. No reports were found in pregnant women.

In adults, the difference in the percentage of goiter among three iodine status areas was significant in one study [37] but not significant in the other study. In the child studies (Table 5), goiter was examined by palpation in seven studies [49–51, 56, 58, 59, 60], rate of visible goiter was described in two studies [41, 55] and ultrasound was used in seven studies [46–48, 52–54, 57]. One study in Chinese children using ultrasound found that goiter rate was significantly higher in the high iodine area than in the control town [46]. In contrast, the other studies did not statistically compare goiter rate. From six studies in Africa [41, 50, 51, 53, 55, 56], goiter rate was distributed widely, from 0% for visible goiters to 86% of goiters diagnosed from thyroid volume to body surface area in ultrasonography. Bimenya *et al*. showed a reduction in goiter rate after the implementation of USI [56], while Seal *et al*. and Henjum *et al*. reported an excess iodine status in refugee camps and a high goiter rate in a high urinary iodine concentration area, respectively [53, 55].

#### Nodules in cross-sectional studies in adults, children and pregnant women

Four cross-sectional studies in adults (Table 4) [35, 37, 40, 42] and two in children (Table 5) [54, 57] evaluated nodules in adults, children and pregnant women. However, results were not consistent: in adults, Du *et al*. showed a significantly high odds ratio in the excess group [35] and Chen *et al*. [40] found no significant difference in adjusted odds ratio, whereas Szabolcs *et al*. reported a significantly low rate in an excess area in elderly people [37]. The two studies in children both described a low rate. [54, 57].

### Biomarkers in cross-sectional studies in adults, children and pregnant women

Among papers in adults, only one showed results for biomarkers of thyroid hormones (footnote of Table 4), namely a significant elevation in TSH in the excess group [36]. In children, nine papers [43–45, 50, 59, 61–64] evaluated biomarkers, of which five also assessed thyroid diseases (Table 6). TSH was significantly elevated in the excess group in half of these studies [44, 45, 50, 63]. Tg is known as a potential biomarker of iodine status [70]. Three studies in children reported significantly higher levels in the excess group than in the adequate group [44, 61, 63]. Zimmermann *et al*. [63] and Shakya *et al*. [61] reported a U-shape curve of Tg from severe deficiency to excess. In pregnant women, five papers described biomarkers [65–69], including three which described biomarkers for both thyroid diseases and thyroid hormones (Table 7). Three studies in pregnant women showed that a higher UIC was associated with a higher TSH [66, 68, 69], while one study in women in all trimesters from the Congo showed that lower UIC (deficient or adequate) was associated with higher TSH [67]. Another of these studies in pregnant women showed significantly higher values in the excess group than in the adequate group [66]. Results for Free T3 (FT3) and Free T4 (FT4) were not consistent among studies, or not significantly different among UIC categories.

From these results, although meta-analysis could not be conducted in some diseases because included studies were few, hyper- and hypothyroidism, goiter and nodule were reported as thyroid diseases under excess iodine status. Especially, SCH was significantly associated with excess iodine intake. Goiter was mainly reported in studies in children. Biomarkers such as TSH and Tg were also mainly reported in children. TSH in the excess group was elevated in half of included studies and a U-shape curve of Tg from severe deficiency to excess was reported in some studies.

## Discussion

This is the first systematic review to evaluate papers which reported excess iodine status. Hyper- and hypothyroidism, goiter and nodule were reported in studies which included excess iodine intake populations. Although diagnostic criteria, degree of excess and source of iodine excess differed among studies of hypothyroidism, SCH in particular was reported to be significantly associated with excess intake as assessed with UIC in several types of studies, including intervention, case-control, follow-up and cross-sectional studies. Allowing that the quality of some of the included papers was low because of the observational design and lack of adjustment analyses, meta-analysis of OH and SCH in adult studies showed a significant increase in excess areas (OR:2.78 and 2.03, respectively).

Generally, although not all patients with SCH progress to OH, some patients with SCH are treated medically [71]. Therefore, reporting the effect of excess iodine intake on SCH is essential, because at least some of the iodine excess intake might be preventable. The mechanism by which an iodine excess induces thyroid diseases is not completely obvious. In most individuals, escape from the Wolff-Chaikoff effect caused by an acute excess occurs due to a decrease in sodium-iodide symporter (NIS) activity [14]. In some individuals in whom high residual NIS activity prevents adaptation to the Wolff-Chaikoff effect, iodine excess induces hypothyroidism [6]. The randomized controlled trial of Sang *et al*. proposed that a total intake of 800 μg/day confers a risk of SCH (supplementation 400 μg, median UIC 672 μg) [20]. Baseline iodine intake was excess in this trial. In the US, UL is 1,100 μg/day, based on the results of an intravenously supplemented trial of iodine which evaluated TSH concentration [4, 72]. Among the case-control studies in this systematic review, UIC in the hypothyroidism case groups was significantly higher than in the control group in some studies but still less than the UL level, at between 179 to 327 μg/L (Table 2, converted from an intake to excretion ratio of 90% [3]). The exception is a study in Korea, in which UIC was extremely high in both the case and control groups [27]. In cross-sectional studies, UIC in the high iodine areas ranged from 338 to 1241 μg/L (Table 4). Since the high iodine areas in many studies showed a UIC which was much higher than the cut-off value of iodine excess, we were unable to clarify the situation in borderline excess areas. In some studies which compared diseases among UIC categories, differences in the prevalence of SCH in categories over 300 μg/L (or 250 μg/L in pregnant women) did not reach statistical significance [43, 67]. Nevertheless, further research is required to make conclusions about marginally excess areas.

Some studies were from countries in which the main source of iodine is considered to be food, namely the USA [60], Japan [38, 57, 69] and Korea [27, 65], while others were from countries where the main sources of persistent excess iodine are water and salt. Therefore, allowing that the studies differed in the inclusion criteria of participants, the low rate (1%) of SCH in Konno *et al*. [38] from Japan might be influenced by the intermittent consumption of excess iodine, notwithstanding the extremely high mean UIC. In addition to the intermittent intake of high iodine-containing foods, two studies which showed an the extremely high UIC used mean instead of median values [27, 42]. Apart from these, Fig 3 shows that many studies in adults, children and pregnant women showed similar results for SCH in our review. Future studies should review the characteristics of vulnerable populations in each group, such as individuals with thyroid antibody. Moreover, they should also study the dose-response relationship, including borderline excess intake, precise mechanisms and susceptibility or preventive factors for SCH.

IIH has been reported in countries in which USI was recently introduced into previously severe iodine-deficiency areas [7, 8]. In our review, only one paper from Zimbabwe reported IIH [39], and this review showed a non-significant increase in hyperthyroidism in areas with a chronic iodine excess. Given that the number of papers about IIH was low, unreported cases should be considered.

Some papers have reported that excess iodine status is due to USI, while other papers showed that an excess iodine status occurred in populations despite low utilization of iodized salt, and main source was determined to be water containing iodine. Goiter is one of the main characteristics of iodine deficiency disorders [4]. Although the effect of excess iodine on goiter in the papers included in this review was controversial, monitoring and maintaining an adequate iodine concentration in salt is essential, given that the goiter rate has decreased in China [24] and Uganda [56] after an increase in UIC following USI implementation. More papers should compare goiter rates between excess and sufficient areas or those before and after USI in one area. The results at this stage are unable to integrate because the assessment of goiter (visible, palpation or ultrasound) and background iodine status differed among studies.

For ethical reasons, a randomized controlled trial to evaluate the threshold for causing thyroid diseases is considered to be difficult. The effect of iodine excess might be better understood by assessing previous papers which examined the safety of the upper limits of iodine, and observational studies in which a population is exposed to chronic excess iodine. However, several limitations of this review warrant mention. First, we only included apparently healthy free-living populations and excluded studies for newborns, assessment of thyroid antibody and the effect of acute excess intake. Individuals with autoimmune thyroiditis seems to be susceptible to high iodine intake [73, 74] and vulnerable sub-populations should be examined in a future review. Regarding confounding factors between excess iodine intake and thyroid diseases, age, sex and positive thyroid antibodies were shown to be associated with thyroid diseases other than iodine intake in the included studies. Even adjusted for age and sex, excess iodine intake and positive TPO antibody were risk factors for SCH independently in Chinese study [31] and interaction effects on the incidence of SCH between high UIC and positive antibody were found in children [44]. However, many studies only identified the crude percentages of the diseases and did not adjust these variables. Therefore, another systematic review for the effect of antibody on thyroid under the iodine excess status should be performed and each study should include multivariate analysis to adjust these variables for establishing the effect of excess iodine intake strictly. Second, our review originally had a problem in reliance because most papers were non-randomized trials and included a high risk of bias, particularly with regard to the adjustment of outcome. Most included papers only described crude percentages. Finally, although funnel plots of cross-sectional studies were not asymmetrical, the number of included studies in each plot was limited, and a risk of publication bias remains. However, considering the difficulty of conducting randomized trials and the lack of any previous systematic review for excess iodine to date, this review has a number of implications for public health administrators. One important message here is the need for monitoring the iodine concentration in local drinking water, and not only the concentration in salt. For researchers, the goiter rate has been mainly assessed in school-age children to date; nevertheless, hypothyroidism should be also carefully monitored in excess iodine areas.

## Conclusion

In conclusion, hyper- and hypothyroidism, goiter and nodule were reported in this systematic review which included studies about excess iodine intake. Although USI improves goiter rate, chronic exposure to excess iodine from water or poorly monitored salt is a risk factor for hypothyroidism in free-living populations. Because of the low quality and limited number of included studies, future well-designed observational studies, especially those reporting adjusted results, are required. Sub-group analyses are also required, including thyroid antibodies.

## Supporting information

S1 Table Database-specific search words. (DOCX)

S2 Table PRISMA 2009 checklist. (DOC)

S3 Table Risk of bias assessment tool. (DOCX)
