# Supplementary material for: Effect of excess iodine intake on thyroid diseases in different populations: A systematic review and meta-analyses including observational studies
Source: PLoS One. 2017 Mar 10;12(3):e0173722. doi: 10.1371/journal.pone.0173722 (PMC5345857; doi:10.1371/journal.pone.0173722)
Supplement: S1 Table — (DOCX) [file pone.0173722.s001.docx]

S1 Additional File Database-specific search words

**PubMed search formula**

(((excess OR excessive OR adverse OR adequa* OR sufficien* OR "more than adequa*" OR "safe upper" OR "tolerable upper" OR replet* OR overload OR "tolerable dose" OR "tolerable dosage" OR "upper limit" OR "wolff-chaikoff" OR "wolff chaikoff" OR escape OR 300 OR 0.3) AND (((iodine OR iodide) AND (diet OR intake OR interven* OR intervention OR supplement OR supplement* OR status OR food OR exposure OR fortif* OR salt OR seaweed OR seafood OR kelp OR kombu)) OR ((iodine OR iodide) AND (urine OR urin* OR concentration OR excret* OR "urine level" OR "UIC*")))) AND ((((iodine OR iodide) AND (urine OR urin* OR concentration OR excret* OR "urine level" OR "UIC*")) OR (thyroid stimulating hormone OR TSH OR thyroglobulin OR thyroglobulin antibody OR thyroxine OR T4 OR triiodothyronine OR T3 OR thyroid stimulating antibody OR thyroid peroxidase OR thyroid peroxidase antibody)) OR (thyroid volume* OR thyroid volume OR thyroid size OR neck palpation OR thyroid ultrasound) OR (hypothyroidism OR thyroiditis OR autoimmune thyroiditis OR hyperthyroidism OR goiter OR thyrotoxicosis OR graves disease[mesh] OR "grave's disease")) AND ((randomized controlled trial OR clinical trial OR intervention study OR "randomized study" OR "randomized studies" OR "randomised study" OR "randomised studies" OR "randomized trial" OR "randomized trial" OR "randomized trials" OR "randomised trial" OR "randomised trials") OR (epidemiologic study OR "observational study" OR "observational studies" OR cohort study OR cohort OR cohorts OR longitudinal study OR prospective study OR follow-up study OR retrospective study OR case-control study OR cross-sectional study OR multicenter study OR multicenter trials OR multicenter trial))) NOT ((animal OR nonhuman OR animal experiment) NOT ((human) AND (animal OR nonhuman OR animal experiment)))

**Medline search words**

| No. | Search Words |
| --- | --- |
| 1 | (iodine$ or iodide$ or iod$).mp. (171082) |
| 2 | (intake$ or interven$ or supplement$ or status or diet$ or food$ or expos$ or fortif$ or salt$ or seaweed$ or seafood$ or kelp$).mp. (3154063) |
| 3 | (urin$ or concentration$ or excre$ or "urin$ level$").mp. (2139741) |
| 4 | 1 and 2 (27159) |
| 5 | 1 and 3 (33049) |
| 6 | (iod$ adj3 (intake$ or status or supplement$ or expos$ or fortif$ or salt$ or diet$ or food$ or seaweed$ or seafood$ or kelp$)).mp. (5311) |
| 7 | 4 or 5 or 6 (51814) |
| 8 | (excess$ or adverse$ or "300" or "0.3" or adequat$ or sufficien$ or "more than adequat$" or "safe upper$" or "tolerable upper$" or replet$ or escap$ or overload$ or "tolerable dose$" or "upper limit$" or wolff-chaikoff$ or "wolff chaikoff$").mp. (1356963) |
| 9 | 7 and 8 (7402) |
| 10 | (thyr?otrop?in$ or TSH or "thyroid stimulating hormone$" or "thyroid-stimulating hormone$" or thyroglobulin$ or tg or thyroxin$ or T4 or triiodothyronin$ or T3 or "thyroid hormone$" or "thyroid stimulating antibod$" or "tyroid-stimulating antibod$" or "thyroid-stimulating immunoglobulin$" or "thyroid stimulating immunoglobulin$" or "thyroid peroxidase$" or thyroidperoxidase or TPO or "anti thyroid peroxidase" or "anti-thyroid peroxidase" or "anti thyroidperoxidase" or "anti-thyroidperoxidase").mp. (178959) |
| 11 | ("thyroid volume$" or "thyroid size$" or "neck palpation$" or ultrason$).mp. (239968) |
| 12 | (hypothyroidism$ or thyroiditis$ or autoimmun$ thyroiditi$ or hyperthyroidism$ or basedow$ or base-dow$ or thyrotoxicosis$ or enlarge$ or goit$ or grave$ or hashimoto$ or autoimmun$).mp. (317633) |
| 13 | 5 or 10 or 11 or 12 (714764) |
| 14 | (randomized$ or randomised$ or randomized controlled$ or controlled clinical$ or intervention$ or clinical trial$).mp. (1607644) |
| 15 | (epidemiolog$ or observation$ or cohort$ or longitudinal$ or follow-up$ or prospective$ or case-control$ or retrospective$ or cross-sectional$ or "cross sectional$").mp. (3206058) |
| 16 | 14 or 15 (4248442) |
| 17 | 9 and 13 and 16 (1610) |
| 18 | limit 17 to ((english or japanese) and humans) (1280) |

**Ichushi-Web and CiNii database**

Iodine AND intake (in Japanese)
