# Supplementary material for: Effect of excess iodine intake on thyroid diseases in different populations: A systematic review and meta-analyses including observational studies
Source: PLoS One. 2017 Mar 10;12(3):e0173722. doi: 10.1371/journal.pone.0173722 (PMC5345857; doi:10.1371/journal.pone.0173722)
Supplement: S3 Table — (DOCX) [file pone.0173722.s003.docx]

S3 Additional File. Risk of bias assessment tool

| Cochrane Collaboration’s tool for assessing risk of bias | Random sequence generation | Allocation concealment | Blinding of participants and personnel | Blinding of outcome assessment | Incomplete  Outcome  data | Selective reporting |
| --- | --- | --- | --- | --- | --- | --- |
| Sang, 2012, Randomized controlled trial [20] | Low  “Computer-generated random numbers” were used | Unclear  Insufficient information | Low  Although blinding of personnel was not written, authors described “Double-blind” | Low  Described “Double-blind” | Low  No missing data | High  Authors measured antibodies but not reported. |
| Thomson, 2011, Randomized controlled trial [21] | Low  “The randomization schemes were generated by using the Randomization website” | Low  “allocation to groups were all carried out by independent researchers” | Low  “The participants, those administering the interventions…were blinded” | Low  “those assessing the outcomes of the interventions were blinded” | High  An error in the manufacture occurred and supplementation was terminated | High  Thyroid hormone values were described only those who developed thyroid diseases. |
| Risk of Bias Assessment Tool for Nonrandomized Studies (RoBANS). | Selection of participants | Confounding variables | Measurement of exposure | Blinding of outcome assessment | Incomplete  Outcome  data | Selective outcome reporting |
| Namba, 1993, Non randomized controlled trial [22] | NA | High  No adjusted analysis | Low  Data obtained from direct measurements | Low  No statement to indicate blinding, but unlikely to affect outcome measurements | Low  No missing data | Low  Expected outcomes were reported |
| Kotwal, 2015, case-control [23] | Low  All were from the same community | High  No adjusted analysis | Low  Data obtained from direct measurements | Low  No statement to indicate blinding, but unlikely to affect outcome measurements | Low  No missing data | Low  Expected outcomes were reported |
| Wang, 2014  case-control [24] | Low  All lived in the same area | High  Adjusted analysis for benign thyroid nodule was not available | Low  Data obtained from direct measurements | Low  No statement to indicate blinding, but unlikely to affect outcome measurements | Low  No missing data | Low  Expected outcomes were reported |
| Du, 2013, case-control [25] | High  No information for participants’ basic characteristics | High  No adjusted analysis | Low  Data obtained from direct measurements | Low  No statement to indicate blinding, but unlikely to affect outcome measurements | Low  No missing data | Low  Expected outcomes were reported |
| Alsayed, 2008, case-control [26] | Low  All lived in the same area | High  No adjusted analysis | Low  Data obtained from direct measurements | Low  No statement to indicate blinding, but unlikely to affect outcome measurements | Low  No missing data | Low  Expected outcomes were reported |
| Kim, 2000, case-control [27] | Unclear  Patients were from one hospital, however controls were unclear | High  No adjusted analysis | Low  Data obtained from direct measurements | Low  No statement to indicate blinding, but unlikely to affect outcome measurements | Low  No missing data | Low  Expected outcomes were reported |
| Ishizuki, 1992, case-control [28] | Unclear  All were patients but whether they were from one hospital or not was not written | High  No adjusted analysis | Low  Data obtained from direct measurements | Low  No statement to indicate blinding, but unlikely to affect outcome measurements | Low  No missing data | Low  Expected outcomes were reported |
| Teng, 2006, follow-up [29] | Unclear  No information for characteristics other than age, sex and historical status of iodine | Low/High  Multivariate Logistic Regression used for limited results | Low  Data obtained from direct measurements | Unclear  No statement to indicate blinding, and information of iodine status might affect thyroid measurements | Low  Follow-up rate in three regions was almost same | Unclear  Outcome was reported in several papers separately |
| Wang, 2015, follow-up [33] | Low  All lived in the same area | High  No adjusted analysis | Low  Data obtained from direct measurements | Unclear  No statement to indicate blinding, and information of iodine status might affect thyroid measurements | Low  Explanation for missing was written | Low  Expected outcomes were reported |
| Aakre, 2015, follow-up [34] | NA | High  No adjusted analysis | Low  Data obtained from direct measurements | Low  No statement to indicate blinding, but unlikely to affect outcome measurements | Unclear  Reasons for missing in the follow-up were written but explanation of missing individuals from baseline to follow-up were not written. | Low  Expected outcomes were reported |
| Du, 2014, cross-sectional [35] | Unclear  Survey period in all areas were not described | Low/High  Multivariate Logistic Regression partially used | Low  Data obtained from direct measurements | Unclear  No statement to indicate blinding, and information about water iodine in towns might affect thyroid measurement | Unclear  No information for the different numbers among results | Low  Expected outcomes were reported |
| Tan, 2014, cross-sectional [36] | Low  All lived in the same county | High  No adjusted analysis | Low  Data obtained from direct measurements | Low  No statement to indicate blinding, but unlikely to affect outcome measurements | Low  No missing data | Low  Expected outcomes were reported |
| Szabolcs, 1997, cross-sectional [37] | Unclear  Participants lived in three nursing homes in different countries and no information for background characteristics of areas | High  No adjusted analysis | Low  Data obtained from direct measurements | Unclear  No statement to indicate blinding, and information of iodine status might affect thyroid measurements | Unclear  Reasons for missing of thyroid measurement in the prophylaxis area were not written (36%) | Low  Expected outcomes were reported |
| Konno, 1993, cross-sectional [38] | NA | High  No adjusted analysis | Low  Data obtained from direct measurements | Low  No statement to indicate blinding, but unlikely to affect outcome measurements | Low  No missing data | Low  Expected outcomes were reported |
| Gomo, 1999, cross-sectional [39] | NA | High  No adjusted analysis | Low  Data obtained from direct measurements | Low  No statement to indicate blinding, but unlikely to affect outcome measurements | Unclear  Reasons for different number of subjects in each measurement were not described | Low  Expected outcomes were reported |
| Chen, 2013, cross-sectional [40] | Low  All lived in the same area | Low  Multivariate Logistic Regression was used | Low  Data obtained from direct measurements | Low  No statement to indicate blinding, but unlikely to affect outcome measurements | Unclear  No description for missing | Unclear  Diseases diagnosed from thyroid hormone values were hardly mentioned |
| Kassim, 2014, cross-sectional [41] | Unclear  Survey area was limited because of insecurity | High  No adjusted analysis | Low  Data obtained from direct measurements | Low  No statement to indicate blinding, but unlikely to affect outcome measurements | Unclear  No explanation for invalid goiter examination | Low  Expected outcomes were reported |
| Henjum, 2012, cross-sectional [42] | NA | Low/High  Multivariate Regression used for limited results | Low  Data obtained from direct measurements | Low  No statement to indicate blinding, but unlikely to affect outcome measurements | Unclear  Explanation of urinary iodine status in missing populations (3%) of water iodine status in regression model was not written | Low  Expected outcomes were reported |
| Nepal, 2015, cross-sectional [43] | Low  All visited clinics in the close geographical area. | Low/High  Multivariate linear regression used for limited results | Low  Data obtained from direct measurements | Low  No statement to indicate blinding, but unlikely to affect outcome measurements | Unclear  Reasons for missing of biomarkers were not written | Low  Expected outcomes were reported |
| Sang, 2013, cross-sectional [44] | Low  All lived in a small geographical area | Low/High  Multivariate Logistic Regression used for limited results | Low  Data obtained from direct measurements | Unclear  No statement to indicate blinding, and information about water iodine in towns might affect thyroid measurements | Low  No missing data | Low  Expected outcomes were reported |
| Gao, 2004, cross-sectional [45] | Unclear  No information for characteristics other than age, sex and historical status of iodine | Low/High  Multivariate Logistic Regression used for limited results | Low  Data obtained from direct measurements | Unclear  No statement to indicate blinding, and information of iodine status might affect thyroid measurements | Low  No missing data | Low  Expected outcomes were reported |
| Lv, 2014, cross-sectional [46] | Low  All lived in a small geographical area | High  Thyroid volume was adjusted by age and BSA but no other adjusted analysis | Low  Data obtained from direct measurements | Unclear  No statement to indicate blinding, and information about water iodine in towns might affect thyroid measurements | Low  No missing data | Low  Expected outcomes were reported |
| Lv, 2012, cross-sectional [47] | NA | High  No adjusted analysis | Low  Data obtained from direct measurements | Low  No statement to indicate blinding, but unlikely to affect outcome measurements | Low  No missing data | Low  Expected outcomes were reported |
| Li, 2012, cross-sectional [48] | High  Outcome was measured only in high iodine area | High  No adjusted analysis | Low  Data obtained from direct measurements | Unclear  No statement to indicate blinding, and information about high iodine area might affect thyroid measurements | High  No detailed explanation for the selection of participants for outcome measurement | Low  Expected outcomes were reported |
| Alsanosy, 2012, cross-sectional [49] | Low  All lived in the same region | High  No adjusted analysis | Low  Data obtained from direct measurements | Low  No statement to indicate blinding, but unlikely to affect outcome measurements | Unclear  No explanation for the response rate of 86% | Low  Expected outcomes were reported |
| Medani, 2012, cross-sectional [50] | Unclear  Participants were randomly selected, but no information on characteristics between participants in high iodine area and those in other areas | High  No adjusted analysis | Low  Data obtained from direct measurements | Unclear  No statement to indicate blinding, and information about high iodine area might affect thyroid measurements | Low  No missing data | Low  Expected outcomes were reported |
| Hussein, 2012, cross-sectional [51] | Unclear  Two areas were in the same country, but no information for characteristics | High  No adjusted analysis | Low  Data obtained from direct measurements | Unclear  No statement to indicate blinding, and information about high iodine area might affect thyroid measurements | Low  No missing data | Low  Expected outcomes were reported |
| Shen, 2011, cross-sectional [52] | Unclear  No information for background characteristics of populations | High  No adjusted analysis | Low  Data obtained from direct measurements | Unclear  No statement to indicate blinding, and information of iodine status might affect thyroid measurements | Unclear  No description for missing | Low  Expected outcomes were reported |
| Henjum, 2010, cross-sectional [53] | Low  All lived in the same area | Low/High  Multivariate Logistic Regression partially used | Low  Data obtained from direct measurements | Low  No statement to indicate blinding, but unlikely to affect outcome measurements | Low  Explanation for response rate was written | Low  Expected outcomes were reported |
| Duarte, 2009, cross-sectional [54] | NA | High  No adjusted analysis | Low  Data obtained from direct measurements | Low  No statement to indicate blinding, but unlikely to affect outcome measurements | Low  No missing data | Low  Expected outcomes were reported |
| Seal, 2005、cross-sectional [55] | NA | High  No adjusted analysis | Low  Data obtained from direct measurements | Unclear  No statement to indicate blinding, and information of iodine status might affect thyroid measurements | Low  No missing data | Low  Expected outcomes were reported |
| Bimenya, 2002, cross-sectional [56] | NA | High  No adjusted analysis | Low  Data obtained from direct measurements | Unclear  No statement to indicate blinding, and information of iodine status in each area might affect thyroid measurements | Low  No missing data | Low  Expected outcomes were reported |
| Ishigaki, 2001, cross-sectional [57] | NA | Low  Multivariate linear regression used | Low  Data obtained from direct measurements | Unclear  No statement to indicate blinding, and information about iodine might affect thyroid measurements | Low  No missing data | Low  Expected outcomes were reported |
| Zhao, 2000, cross-sectional [58] | Unclear  All lived in the same province but in three counties and background characteristics of populations and survey period were unknown | High  No adjusted analysis | Low  Data obtained from direct measurements | Unclear  No statement to indicate blinding, and information of iodine status might affect thyroid measurements | Low  No missing data | Low  Expected outcomes were reported |
| Boyages, 1989, cross-sectional [59] | Unclear  No information other than iodine content of drinking water was written in two areas | High  No adjusted analysis | Low  Data obtained from direct measurements | Low  No statement to indicate blinding, but unlikely to affect outcome measurements | Low  No missing data | Low  Expected outcomes were reported |
| Trowbridge, 1975, cross-sectional [60] | Unclear  Participants were from four areas | High  No adjusted analysis | Low  Data obtained from direct measurements | Unclear  No statement to indicate blinding, and information of goiter status might affect thyroid measurements | Low  No missing data | Low  Expected outcomes were reported |
| Shakya, 2015 cross-sectional [61] | Low  Two areas were in a small geographical area | High  No adjusted analysis | Low  Data obtained from direct measurements | Low  No statement to indicate blinding, but unlikely to affect outcome measurements | Low  No missing data | Low  Expected outcomes were reported |
| Zou, 2014, cross-sectional [62] | Low  All lived in the same area | High  No adjusted analysis | Low  Data obtained from direct measurements | Low  No statement to indicate blinding, but unlikely to affect outcome measurements | Unclear  No reasons are given for the different numbers among measurements | Low  Expected outcomes were reported |
| Zimmermann, 2013, cross-sectional [63] | Unclear  Countries and areas were selected to provide varying regional, ethnic representation but no information on these in each UIC category | High  No adjusted analysis | Low  Data obtained from direct measurements | Low  No statement to indicate blinding, but unlikely to affect outcome measurements | Low  No missing data | Low  Expected outcomes were reported |
| Zimmermann, 2005, cross-sectional [64] | High  Participants lived in five different countries and no information for background characteristics of areas | Low  Multivariate linear regression used | Low  Data obtained from direct measurements | Low  No statement to indicate blinding, and information about high iodine area might affect thyroid measurements, but intra-and inter-observer variability were written | Low  No missing data | Low  Expected outcomes were reported |
| Cho, 2015, cross-sectional [65] | NA | High  No adjusted analysis | Low  Data obtained from direct measurements | Low  No statement to indicate blinding, but unlikely to affect outcome measurements | Low  No missing data | Low  Expected outcomes were reported |
| Shi, 2015, cross-sectional [66] | Low  All lived in the same area | Low/High  Multivariate Logistic Regression partially used | Low  Data obtained from direct measurements | Low  No statement to indicate blinding, but unlikely to affect outcome measurements | Low  No missing data | Low  Expected outcomes were reported |
| Habimana, 2014, cross-sectional [67] | Low  Participants in each category selected equally from three areas and three trimesters | Low/High  Multivariate Logistic Regression used for limited results | Low  Data obtained from direct measurements | Low  No statement to indicate blinding, but unlikely to affect outcome measurements | Low  No missing data | Low  Expected outcomes were reported |
| Sang, 2012, cross- sectional [68] | Unclear  Two areas were in the same country, but no information for characteristics | High  No adjusted analysis | Low  Data obtained from direct measurements | Unclear  No statement to indicate blinding, and information about high iodine area might affect thyroid measurements | Low  No missing data | Low  Expected outcomes were reported |
| Orito, 2009, cross-sectional [69] | Low  All were pregnant women who visited the same hospital | High  No adjusted analysis | Low  Data obtained from direct measurements | Low  No statement to indicate blinding, but unlikely to affect outcome measurements | Low  Explanation for missing was written | Low  Expected outcomes were reported |
